# Design and Implementation of a Multidimensional Visualization Reconstruction System for Old Urban Spaces Based on Neural Networks

**DOI:** 10.1155/2022/4253128

**Published:** 2022-06-02

**Authors:** Shuhua Wang, Anhua Qin

**Affiliations:** ^1^Landscape Architecture, Zhejiang Gongshang University, Hangzhou 310000, China; ^2^Landscape Architecture, Zhejiang Sci-Tech University, Hangzhou 310000, China

## Abstract

This article presents an in-depth study and analysis of the construction of a convolutional neural network model and multidimensional visualization system of old urban space and proposes the design of a multifaceted visualization reconstruction system of old urban space based on a neural network. It also quantitatively analyzes the essential spatial attribute characteristics of urban shadow areas as nodes of the overall urban dynamic network in three dimensions—spatial connection strength, spatial connection distance, and spatial connection direction—summarizes the characteristics of urban old spatial structure from the perspective of a dynamic network, and then proposes the model of urban old spatial design from the perspective of an active network. The shallow depth of the network structure is used to reduce the parameters in the learning process of reconfigurable convolutional neural networks using data sets so that the model learns more general features. For the situation where the number of data sets is small, data augmentation is used to expand the size of the data sets and improve the recognition accuracy of the reconfigurable convolutional neural network. A real-time update method of multifaceted data visualization for big data scenarios is proposed and implemented to reduce the network load and network latency caused by charts of multidimensional data changes, reduce the data error rate, and maintain the system stability in the old urban space concurrency scenario.

## 1. Introduction

Since the reform and opening, cities have been in rapid development due to the need for economic construction. Although the rough development mode has been adapted to the rapid growth of urban development, it has also led to the problem of “urban disease” in some cities. Traditional urban renewal has caused damage to the urban fabric, fragmentation of urban space, loss of urban landscape, and fracture of historical heritage [1]. To avoid wasting resources, the total growth of new buildings has started to slow down, which has forced urban construction to shift from incremental planning to stock optimization. The so-called “stock optimization” maintains the existing total amount of building land based on non-expansion and development and optimizes and upgrades the existing urban space to promote organic urban renewal [2]. The main target of stock optimization is the old urban areas, including urban villages, senior communities, shantytowns, and other urban spaces with decaying physical environments. Using the means of organic regeneration, we can reasonably plan the existing stock resources, improve the living environment of community residents, promote the optimization of community functions, introduce a variety of commercial forms, and use the original buildings to implant new urban parts, to realize the double improvement in community living standards and economic benefits. Compared with incremental development, urban stock optimization activates urban public space through regeneration to optimize the physical aspect of urban space, maximize land value, promote residents' quality of life, and perpetuate the cultural spirit contained in the metropolitan area from the nonmaterial cultural part.

With the development of artificial intelligence, various convenient and intelligent devices have been widely used in people's daily production and life. Smart devices for road risk target detection have provided people with convenience. Alpha Go's victory over Go reawakened the world, and convolutional neural networks came back to the forefront. The complex networks with many nodes and certain connections between nodes in the real world can be abstracted as graph model [3]. In response to the problem that traditional deep learning algorithms cannot perform convolutional operations on graph data, researchers have designed graph neural networks (GNNs) for processing graph data. The weak changes in the underlying network parameters, which are transmitted through layers of linear transformations and nonlinear activations, are amplified layer by layer deep in the network, resulting in changes in the input distribution at each layer, and the deep network needs to constantly adapt to these distribution changes. Graph neural networks are becoming increasingly popular in various fields, including knowledge graphs, traffic networks, social networks, and even life sciences, and the power of GNNs in modeling dependencies between nodes in graphs has led to breakthroughs in research areas related to graph analysis [4]. With the above information fusion advantages, heterogeneous network analysis has rapidly become a research hotspot in data mining, database, and information retrieval, comprehensively involving various basic tasks, such as classification, clustering, recommendation, etc. With the rise of network representation learning, heterogeneous network representation learning has also rapidly stimulated the interest of a wide range of researchers, and learning to obtain low-dimensional vector representations of graphs can also improve performance while accelerating downstream tasks.

In the information-advanced data era, people collect and apply more and more data in various aspects. Various massive and complex data sets are generated to extract valuable information for individuals, enterprises, and society from the data, which become new sources of value [5]. Multidimensional data, an essential component of data sets, is now spread across different domains, and information is mainly accessed through several other sources. The creation of big data has led to extensive research in data visualization techniques, which mainly focus on the analysis and statistics of large amounts of data to predict trends. To communicate information more clearly, achieve graphical presentation, and perfect communication of critical data features, data visualization techniques must simultaneously balance the essence of aesthetic form and function, provide deep insight into multi-scene data sets, and complete communication between people and information [6]. Data visualization includes scientific information and visual analytics, converting abstract data into easily recognizable graphical or pictorial information and interactive processing, providing a quantitative basis for better, faster, and sustainable Internet development. As a research area under information visualization technology, multidimensional data visualization focuses on how to render multidimensional data into human-understandable two-dimensional or three-dimensional graphic images through various methods. For these multidimensional massive data sets, firstly, some complete multidimensional data visualization systems are not suitable for multi-scene applications and are only used for visual analysis of specific data types, with poor generality; secondly, multidimensional data are variable and do not provide good support for real-time updating of data charts; finally, layered processing of multidimensional data sets is not supported in general visualization platforms, and nonprofessional users can only perform a simple. This limits the application of multidimensional data visualization technology to a certain extent [7]. In the face of massive and changeable multifaceted data sets, there are still poor systematization, management, and generality of data visualization; lack of data function support; and limited multidimensional data research in the existing visualization system.

## 2. Related Works

In recent years, algorithms based on deep learning have dominated various fields of computer vision. Convolutional neural networks have been introduced into remote sensing image classification due to their powerful feature extraction ability. Research has been carried out on high-score remote sensing classification methods based on convolutional neural networks for rural building information extraction. Erol B used an object-oriented segmentation technique to segment UAV images and then used convolutional neural networks combined with sliding windows for building detection to achieve building information extraction of hollow villages based on UAV high-score photos [8]. Motta et al. used Seg Net semantic segmentation model and World View-2 high-resolution images to extract rural buildings in City, Hebei Province [9]. They achieved an overall accuracy of up to 96% compared to learning algorithms such as support vector machines and random forests. Existing studies show that the deep learning-based method of extracting rural settlement buildings from high-definition remote sensing has obvious advantages with the support of many samples. However, the data preprocessing stage still relies on the manual annotation to generate training samples, with a high time cost [10]. On the other hand, most existing studies directly apply the convolutional neural network models in natural image recognition to remote sensing. The deep models constructed for the characteristics of remote sensing images or classification objects still need further research.

The object to be visualized is 3D body data, and the corresponding visualization algorithm is the body drawing algorithm. Instead of generating intermediate metadata, body drawing calculates screen pixel point values based on the contribution of each data point in the 3D data field to the 2D image. Currently, there are standard body drawing methods such as ray projection, texture mapping, staggered cut, footprint table, etc. Among them, the ray projection method was proposed by Kuan in 1988 and gained some development and application, which is a classical body drawing method. Then Cheng perfected this algorithm by opacity early cutoff; Phelps optimized it based on the data storage structure of octree. And with the development of graphics hardware GPU, various body drawing algorithms have been accelerated. For example, Thomas et al. took the splatting algorithm as the research object and achieved high-quality image drawing by improving the light simulation model in the algorithm process and accelerating the GPU hardware according to the spherical harmonic illumination theory [11]. Williams et al. use octree coding and GPU parallel computing framework for the body rendering process of texture mapping. The body rendering algorithm can be used to generate, map, and synthesize textures in hardware. The rendering effect and efficiency can be improved by using GPU graphics hardware to support 2D or 3D surfaces [12]. For example, Zhang et al. used graphics hardware to provide the effect of light and dark gradients for the algorithm; Gao et al. used GPU to complete the reconstruction of objects and arbitrary background fusion to achieve the method of environmental texture mapping [13]. Simon et al. applied GPU technology to 3D texture body drawing and used semitransparent light and dark processing algorithm to improve the drawing efficiency of body drawing [14].

Standard methods of multidimensional data visualization techniques include parallel coordinates, scatterplot matrix, radial coordinates, etc. Scholars' research on multidimensional data visualization mostly complements and improves the existing visualization techniques [15]. Wang proposed a multi-view collaborative analysis method based on parallel coordinates, which embeds histograms in parallel coordinates and performs visual analysis of the same data set in collaboration with scatterplot matrices, integrating the advantages of different visualization techniques and making the local details of data more clearly presented [16]. Buhrmester proposed uncertainty visualization and analysis methods for the uncertainty problem in the visualization process and carried out relevant research around modeling, metrics, visualization, and circumvention of uncertainty in multidimensional data [17]. Cao et al. seamlessly integrated parallel coordinates with scatterplots and researched and developed the SPPC (scattering points in parallel coordinates) visualization tool, which realizes the human-computer interaction function on parallel coordinates [18]. El Sayad and Ayad proposed scaling-independent multidimensional data visualization in two-dimensional space, which can visually present changes in multifaceted data in a single window and allows users to explore the data at different scaling levels interactively [19]. Cai represented data dimensions as entity attributes to eliminate data redundancy and then proposed a scalable, dynamic visualization model to display different levels of detailed information to generate effective visualization results [20].

## 3. Neural Network Model Construction

Convolutional neural networks are mainly used to process data with multidimensional array structures, such as one-dimensional language sequences, two-dimensional image and audio mapping, and three-dimensional natural signals such as video [21]. The standard components for building convolutional neural networks are mainly the convolutional layer, pooling layer, activation layer, batch normalization layer, fully connected layer, an output layer, etc.

**Figure 1:**
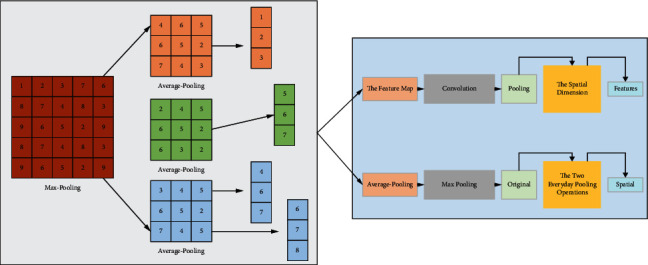
Pooling operation.

(1) Convolutional layer: The convolutional layer is the primary building block of a convolutional neural network. A convolutional layer consists of several convolutional kernels (neurons) with learnable parameters. According to the weight sharing theory, the underlying features of an image are independent of the location, that is, the elements learned in one local area can be used for feature extraction in other local areas. Therefore, the convolutional layer uses convolutional kernels with fixed weights to generate local features by sliding the local regions of the input image, which is called regional connectivity. The size of each convolution kernel is called the receptive field. It represents the size of the area corresponding to each pixel in the upper layer of the output feature map. Due to the fixed weights, each convolution kernel focuses on a single image feature, such as edge, color, or texture. The use of weight sharing and local connectivity can effectively reduce the number of parameters for network training, making the operation concise and efficient. The process of convolution operation can be expressed as follows:
  (1) $$\begin{array}{c} x_{ijk}=\sum_{k=1}K+\sum_{m=1}m+\sum_{n=1}\left(n+1\right). \end{array}$$
(2) Pooling layer: The pooling layer is generally used to down sample the feature map output from the convolution operation in the spatial dimension. The two everyday pooling operations are Max pooling and Average pooling, as shown in Figure 1. Max pooling samples the maximum value of the region to be pooled, while average pooling calculates the average value of the input features. The role of the pooling layer is to reduce the dimensionality of the data using statistical methods to minimize redundant information and retain the most scale-invariant features. After pooling layer sampling, although the spatial resolution is reduced, the pooled region corresponds to a more extensive range of the original image, increasing the perceptual field of the convolutional neural network and improving the network's robustness in extracting features. The pooling layer does not introduce additional training parameters and only reduces the size of the feature map.
(3) Activation layer: The convolutional layer is a linear weighted summation for the feature extraction process, and the superposition of multiple convolutional layers is a linear transformation. It is necessary to use a nonlinear activation function to perform a nonlinear mapping of the output of the convolutional layer. By using alternating convolutional and activation layers, the nonlinear characteristics of the neural network are increased, which makes the model have enough ability to approximate arbitrary functions. The activation functions commonly used in convolutional neural networks include the sigmoid, tanh, and linear rectification (ReLU).
The sigmoid function maps the number taking the value of (–*∞*, +*∞*) between (0, 1), which can be used in the network's last layer for the binary classification function. Sigmoid function as a nonlinear activation function in the convolutional neural network is not often used and mainly has the following drawbacks: (1) When the value of *z* takes values beyond the range of [–6, 6], the function value changes almost invariably, and the corresponding derivative *g*′(*z*) is close to 0, which means that the gradient of the weight *w* is close to 0 and difficult to update, that is, the gradient disappearance problem; (2) The sigmoid function is not asymmetric origin function, and its output is always positive, making the optimization path of the gradient in the backpropagation process of network training prone to jagged oscillations, increasing the convergence time. The derivation of the sigmoid function is derived as
  (2) $$\begin{array}{c} \;g^{\prime }\left(z\right)=\sum \left(\frac{e–1}{\sqrt{e^{z}–1}}\right). \end{array}$$
The tanh function is like the sigmoid function in that it can be considered linear when *z* takes values in the range around 0. Compared with the sigmoid function, the tanh function has a mean value of 0, which makes up for the shortcomings of the non-origin symmetry of the sigmoid function. However, when *z* values are taken beyond a specific range, the process's derivative *g*′(*z*) is close to 0 most of the time, and the same gradient disappearance problem will occur. The function derivation is derived as
  (3) $$\begin{array}{c} g_{\left(z–1\right)}=\sum_{z=1}\frac{e^{z}}{\sqrt{e^{z}–e^{–z}}}. \end{array}$$
The ReLU function does not have a gradient disappearance problem when the input is positive, and the linearity of the function makes the forward and backward propagation fast. However, when the information is negative, the derivative *g* is constantly equal to 0, and the gradient disappearance problem will arise. The derivative of the ReLU function is
  (4) $$\begin{array}{c} g=\sum_{z=1}\frac{\sqrt{z–1}}{z}. \end{array}$$
(4) Batch normalization layers: During the training process of the convolutional neural network, the parameters in the network are constantly updated through gradient descent and back propagation, and there is a high degree of correlation and coupling between layers in the network. The weak changes in the underlying network parameters are passed through layers of linear transformations. Nonlinear activations are amplified layer by layer in the deeper part of the network, resulting in changes in the input distribution of each layer, and the deeper network needs to constantly adapt to these changes in distribution; on the other hand, due to the saturation of the activation function, the distribution of the output features of each layer will gradually approach the upper and lower ends of the activation function, making the training process easy to fall into the gradient saturation zone, where the gradient becomes small and close to 0, making the model training difficult. The above phenomenon is called internal covariate shift. Subsequent studies have proposed a batch normalization layer. Batch normalization (BN) normalizes the input data of each batch during the network training process so that the feature distribution of each set has the same mean *μ* and variance. The following equation can express the process of batch normalization.
  (5) $$\begin{array}{c} y_{i}=\int \left(r–\frac{x_{i}–u_{i}}{\sqrt{a_{i}–e}}–\beta \right). \end{array}$$
(5) Fully connected layer: The fully connected layer maps the feature representations extracted by the neural network to the sample labeling space and plays the role of classification. Since the neurons of the fully connected layer take all connections between layers, the parameters of the fully connected layer usually account for more than 50% of the parameters of the whole network. The output of the fully connected layer is in vector form, leading to the loss of structural information. The input size that a convolutional neural network can accept is usually limited by the parameters of the fully connected layer. The main difference between a fully convolutional neural network (FCN) and a convolutional neural network (CNN) is that the FCN transforms the fully connected layer at the end of the CNN into a convolutional layer.
(6) Output layer: At the top of the convolutional neural network, an output layer is set to output the posterior probabilities of each category. A typical output layer is SoftMax, which is often used in the multi-classification process to map the previous layer's output to the (0, 1) interval, which can be regarded as outputting the posterior probabilities for each category with the sum of all categories being 1. For the *k*-classification problem, the value of SoftMax for each category is
  (6) $$\begin{array}{c} w=\sum_{i=1}m\left[x^{i}\left(\frac{y^{i}+j}{y–j}\right)\right]. \end{array}$$
Fully convolutional networks can be applied to semantic segmentation tasks. Compared with CNN, FCN is an end-to-end network model that uses a 1 × 1 convolutional layer to replace the fully connected layers in CNN and up samples the feature map with the same size as the input image to perform pixel-by-pixel classification of the input image [22]. The FCN model and its extensions and improvement methods have become mainstream in semantic segmentation. Many modular structures for the characteristics of semantic segmentation tasks have emerged in the application to improve classification accuracy.
(1) Encoder-decoder architecture: The encoder-decoder network architecture mainly consists of an encoder module for acquiring abstracted semantic information layer by layer, accompanied by a reduction in the size of the feature map, and a decoder module for recovering the spatial resolution of features by up sampling layer by layer. The encoder module usually uses CNNs with fully connected layers removed (e.g., VGG, Res Net, etc.) as feature extractors to extract abstracted deep features top-down through a primary sequence of convolution-activation-pooling. In this process, the size of the output feature map is smaller than the original input, and spatial detail information is inevitably lost due to the inclusion of a down sampling operation. The decoder usually adopts an asymmetric structure with the encoder, up samples the deep semantic features extracted by the encoder layer by layer from the bottom-up, and then outputs the semantic segmentation result with the original graph size. In addition, to recover the spatial information lost in down sampling during encoder feature extraction, a skip connection is often used in the decoder stage to superimpose and fuse the deep low-resolution feature map with the shallow, high-resolution feature map or use the pooled index values saved during down sampling to recover the up sample. Standard FCN models of encoder-decoder structure include Seg Net, U-Net, etc.
(2) Dilated convolution layer: pooling or cross-step convolution (step size greater than 1) in convolutional neural networks and other operations to down sample features, in the extraction of features while expanding the field of perception. However, the loss of spatial information will affect the semantic segmentation of pixel-by-pixel prediction, without the use of pool layer and other down sampling operations. Dilated convolution injects holes (zero values) into the standard convolution kernel to increase the receptive field. Compared to the regular convolution layer, dilation convolution introduces a dilation rate parameter, which refers to the interval between each convolution kernel element. The perceptual field of the dilation convolution is calculated as follows:

(7) $$\begin{array}{c} r_{n}=r_{n+1}–\sum d\left(k_{n–1}\right). \end{array}$$

Using dilation convolution instead of pooling operation can avoid the loss of more input signals while increasing the perceptual field so that the output of each convolutional layer contains a more extensive range of information, which to some extent resolves the conflict between maintaining the spatial resolution of features and increasing the perceptual field of the network. MOLAP can be used to realize precomputation of multidimensional data Cube, query on the basis of precomputation, quickly return query results according to user input, store both detailed data and aggregated data in Cube cubes, and improve spatial conversion rate and query efficiency through multidimensional techniques such as rotation, slicing, and dicing. On the other hand, the dilation convolution layers with different dilation rates correspond to obtaining feature information at different scales. Standard FCN models using dilation convolution are the Deep lab series.

## 4. Design of Multidimensional Visualization Reconstruction System for Old Urban Space

**Figure 2:**
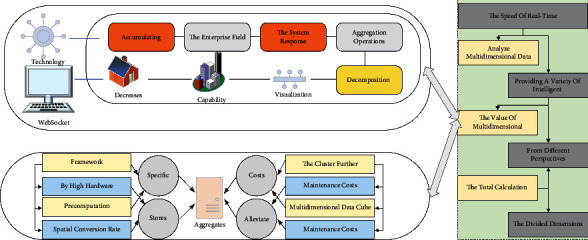
Flow chart of the visualization method for multidimensional data.

The multidimensional data have been accumulating with the increase in the enterprise field, which brings tremendous pressure to the database and makes the system response speed very slow in querying data and aggregation operations. To solve the problem of deep decomposition of multidimensional data and efficient visualization and analysis of multidimensional data, the real-time computing capability is generally improved through distributed clusters to speed up each query. However, with a specific cluster size, the query performance decreases dramatically as the volume of multidimensional data increases sharply. Multidimensional data are constantly updated; different users need to perform multidimensional data analysis in this system, which leads to the need for clients to send data to the server concurrently. In order to verify whether the high concurrency and stability of the system server meets the standard under the situation of frequent data requests, the Jmter test tool is mainly adopted to set different numbers of concurrent connections, respectively, and the average response time is derived according to the number of concurrencies. One way to alleviate the problem is to expand the cluster further, accompanied by high hardware and maintenance costs. Therefore, this system uses MOLAP to implement precomputation of multidimensional data Cube, query based on precomputation, return query results quickly according to user input, store both detailed data and aggregated data in Cube cubes, and improve spatial conversion rate and query efficiency through multidimensional techniques such as rotation, slicing, and dicing [23]. Based on the above analysis, this system designs and implements a visualization framework for multidimensional data, which helps achieve more effective collaborative updating of data charts in complex and thousands of data sets. Firstly, the Kylin plug-in defines the data model for the data set and builds Cube cubes, aggregates the divided dimensions and metrics, and stores the user-built multidimensional Cube cubes in HBase for analysis. Secondly, to avoid the total calculation of Cube for multidimensional data, Kylin is used to realize incremental computation and storage of Cube, thus increasing the speed of real-time querying multidimensional data. Finally, Echarte technology is used to visualize and analyze multidimensional data, providing a variety of intelligent charts to explore the value of multidimensional data from different perspectives. WebSocket is used for real-time pushing to realize real-time updates of multifaceted data, and Kanban is used when a data change is detected. The overall flow chart is shown in Figure 2.

**Figure 3:**
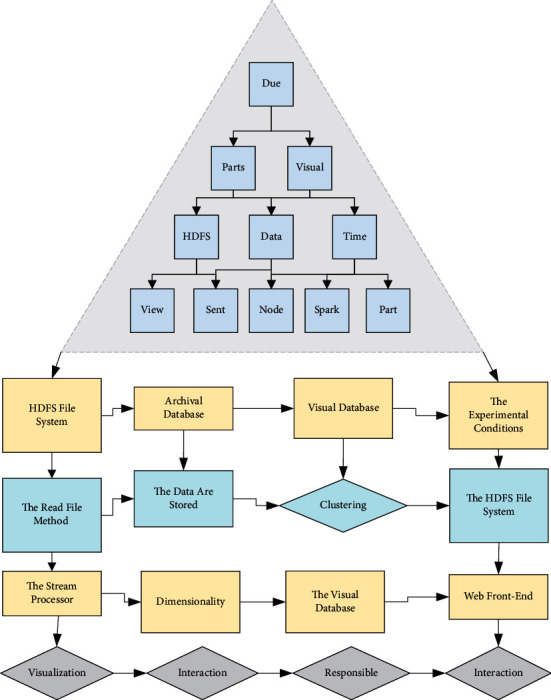
System architecture diagram.

According to the multifaceted stream data visualization and analysis process, the prototype system architecture can be divided into three parts, namely data storage, processing, and visualization, and is shown in Figure 3.

**Figure 4:**
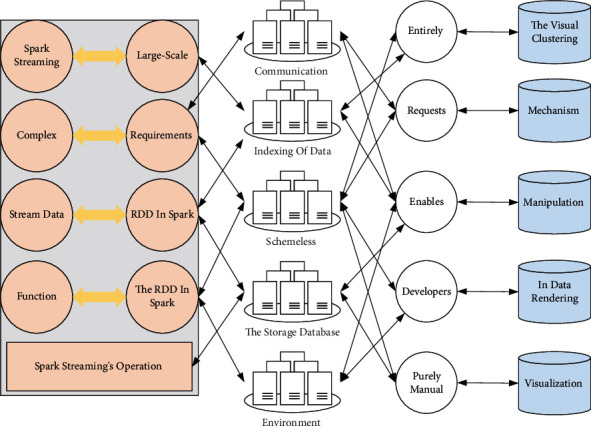
Visual analysis flow chart.

1. There are three parts of data storage: HDFS file system, archival database, and visual database. Due to the limitation of the experimental conditions, this article uses the read file method to simulate the streaming data, and all the data are stored in the HDFS file system; when the multidimensional streaming data arrive at the stream processor (Spark Streaming), the processor will save all the data to the archive database according to the time sequence; the visual database stores the data that need to be visually analyzed, that is, the result set after streaming data clustering The visualization database stores the data for visual analysis, that is, the result placed after clustering and dimensionality reduction.
2. The data processing part has two servers: One is the stream processor, Spark Streaming stream data, for data clustering and dimensionality reduction and then stored separately; and the second is the Node server, which is mainly responsible for dynamic data querying of the visual database and communication with the web front-end.
3. The data visualization part is divided into two modules: The visualization mapping module and the user interaction module. The visualization mapping module is responsible for mapping the stream data set sent from the Node server side to the corresponding visualization view (parallel coordinates, radial coordinates, etc.); the user interaction module communicates with the server side according to the user's request, dynamically adjusts the data model and the visualization model, and finally gets the visualization view required by the user.
4. The multidimensional streaming data are acquired dynamically and in real time, which requires a certain temporal coherence of the visualization results and requires the data mining methods and visualization methods to be efficient enough to give real-time feedback. Therefore, in this article, Spark, a distributed platform, is chosen to improve the computing efficiency of the system, and a browser is selected as the visualization output of the system. The specific process of visual analysis is shown in Figure 4.
5. Streaming processor Spark Streaming: Spark Streaming uses the concept of micro-batch for data mining, which can cope with large-scale complex multidimensional stream data and meet the real-time requirements of visual analysis of stream data. Data augmentation techniques, reduction of the number of convolutional neural network layers, and loss functions are applied. The well-built reconfigurable convolutional neural network model can be trained on small data sets and completed based on small-scale data sets. By setting a time window, the stream data are split into discrete streams in the form of Streams. Each Stream can be transformed into an RDD in Spark, and Spark Streaming's operation on the Stream can be transformed into a process on the RDD in Spark, and the function on the RDD is saved to the memory [24]. The multidimensional stream data clustering and dimensionality reduction algorithms proposed in this article are based on Spark Streaming for parallelization implementation.
6. MongoDB database: The mainstream data format used in today's front and backend web communication is JSON. To facilitate the storage and indexing of data, this system uses the schemaless document storage system MongoDB—the storage database of visual data.
7. Web Backend-Node and Express framework: Node is a server-side runtime environment based on Google Chrome's V8 engine, using a single thread to run. Node uses an event-driven callback style based entirely on a non-blocking I/O library so that the server side can cope with large-scale connection requests. Express is a framework on Node that enables developers to do web development faster.
8. Web front-end-D3: *js* purely manual writing of visualization scripts can be tedious, boring work, and very inefficient in data rendering. Data-driven-documents (D3) provides a rich set of visual manipulation primitives that are used to simplify most of the computations in rendering. In addition, it provides a data binding mechanism that allows developers to build visualization views and user interaction modules quickly [25]. The visual clustering and radial coordinate mapping optimization algorithms for multidimensional streaming data proposed in this article are implemented using D3.

## 5. Analysis of Results

### 5.1. Neural Network Model Analysis

**Figure 5:**
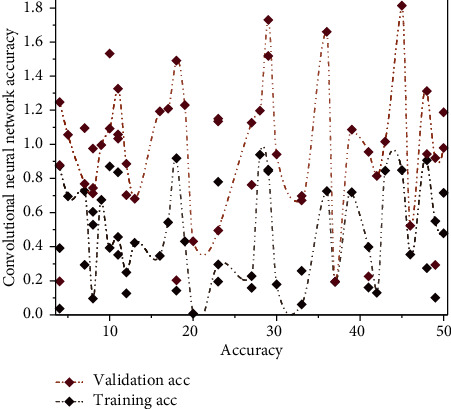
Accuracy of the convolutional neural network before joining.

Based on the methods mentioned above to improve the convolutional neural network model, this study will design a shallow reconfigurable convolutional neural network model with five convolution and pooling operations layers. In addition, to solve the problem of improving recognition accuracy and reducing overfitting in a reconfigurable convolutional neural network with a small amount of data, data enhancement technology reduces the number of layers of convolutional neural network and applies loss function. The reconfigurable convolutional neural network model can be trained on small data sets. Finally, a convolutional neural network model with low loss and about 95% accuracy is obtained. The convolutional neural network model constructed in this article requires the input images to be preprocessed first, and the batch size of the photos is set to 150 × 150. The convolutional neural network without data enhancement and regularization methods is trained separately from the convolutional neural network with reduced overfitting. The preprocessed data set is used as input to compare the effect of data enhancement and regularization methods on the recognition accuracy and loss of the convolutional neural network in the case of different data sets. The accuracy of the convolutional neural network before joining is shown in Figure 5.

The red curve shows the performance when training the convolutional neural network, and the blue curve shows the performance when testing the convolutional neural network. The vertical coordinate represents the model's accuracy. The horizontal coordinate represents the number of image traversals in the data set and the accuracy performance of the reconfigurable convolutional neural network on the training and test sets. Training (training accuracy) is red, and validation accuracy (factual accuracy) is blue. The recognition accuracy of the reconfigurable convolutional neural network is close to 100% as the training proceeds when it utilizes the data on the training set, which indicates that the convolutional neural network can fully recognize the information on the training set.

In contrast, the performance of the reconfigurable convolutional neural network on the actual test set shows that the recognition fluctuates more as the test proceeds and eventually converges to approximately 92% accuracy. The difference in performance on the two data sets is more pronounced, with a significant difference in inaccuracy [26]. When the reconfigurable convolutional neural network is tested using the image data in the training set, the loss gradually decreases during the training process and eventually tends to 0. Real-time monitoring of data and changes in data sources cause the visualization charts to be updated automatically, solving the problem of unchanging charts in the system due to multidimensional data changing anytime and anywhere, reducing the steps of redrawing charts for data changes in older urban spaces. The loss tends to fall from the beginning when the reconfigurable convolutional neural network is tested using the test set. Still, it soon remains constant at about 0.2 and increases as the experiment progresses.

**Figure 6:**
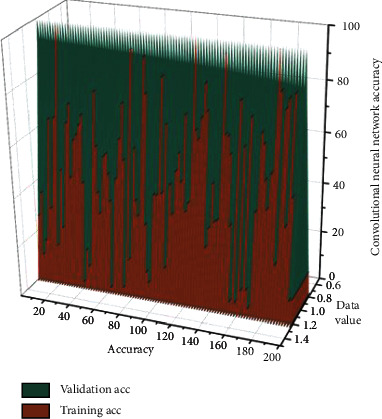
Accuracy of the convolutional neural network after joining.

Moreover, the data sets used in the above two cases still contain more image data. Suppose we continue to reduce the number of images in the training and test sets. In that case, the accuracy of the reconfigurable convolutional network before adding data enhancement and regularization methods will be lower and the loss will be higher. The reconfigurable convolutional network will perform worse on the whole test set. The red curve shows the performance when training the convolutional neural network, and the blue curve shows the performance when testing the convolutional neural network. By completing the deployment of the prototype system for old urban spaces in the Jetson Nano module component, image recognition can begin. The vertical coordinate represents the model's accuracy, and the horizontal coordinate represents the number of image traversals in the data set. The accuracy of the added convolutional neural network is shown in Figure 6. Training accuracy is in red and validation accuracy is in blue. As can be seen, the reconfigurable convolutional neural network performance on the two data sets gradually approaches, and the accuracy continues to improve as the training and recognition process proceeds. This can indicate that the recognition accuracy of the shallow reconfigurable convolutional neural network model can be effectively improved by adding the data enhancement and regularization methods. The failure of the reconfigurable convolutional neural network on the training set performs approximately the same as when the data enhancement and regularization methods are not added. This indicates that the inclusion of such practices does not impact the training process of the reconfigurable convolutional neural network. While the curve on the test set fluctuates more, the overall angle gradually decreases and converges to the loss profile on the training set as the test proceeds. Compared to the reconfigurable convolutional neural network before the data enhancement and regularization methods were added, the loss situation is significantly reduced.

Comparative experiments are performed on shallow reconfigurable convolutional neural networks before and after adding data enhancement and regularization methods using identical training and test sets [27]. The comparison shows a significant improvement in the accuracy of the reconfigurable convolutional neural network, and on top of that, the loss situation is significantly reduced. The reconfigurable convolutional neural network will perform better as the number of data increases and the experiments continue.

### 5.2. Implementation of Multidimensional Visualization Reconstruction System for Old Urban Space

**Figure 7:**
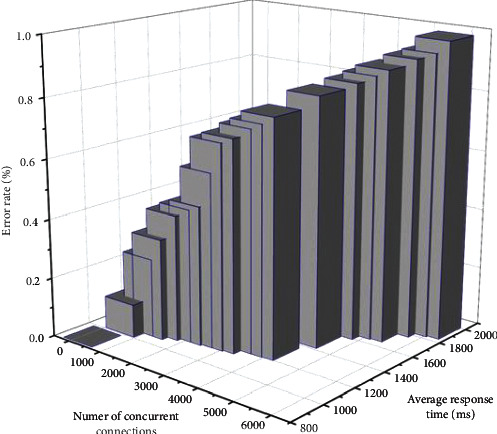
Testing of concurrent connections and average response time.

The performance test for the system mainly lies in testing the response time required by the user to use the system. In this article, we take the real-time update function in the cockpit as an example; for the built multidimensional visualization system, on the one hand, the application data are multidimensional, and with time, the multidimensional information is constantly updated; and on the other hand, different users need to analyze multidimensional data in this system, which leads to the need for the client to send data to the server concurrently, to verify that in the case of frequent requests for data, the system server meets high concurrency. To confirm whether the high coexistence and stability of the system server meet the standard under the frequent data requests, we mainly adopt JMeter test tool to set different concurrent connections and derive the average response time according to the concurrency number. When the number of simultaneous connections exceeds 900, the error rate is 0. All the data are stored in the HDFS file system; when the multidimensional stream data arrive at the stream processor (Spark Streaming), the processor saves all the data to the archive database in chronological order; the visualization database stores the data that need to be visually analyzed, that is, the result set after clustering and dimensionality reduction of the stream data. When the number of connections reaches 1500, the error rate is about 0.83%, which means that the system's performance can meet the requirements of the actual multi-application multi-tenant mode of time data push, thus achieving good stability practical applicability. The test of concurrent connections and average response time is shown in Figure 7.

**Figure 8:**
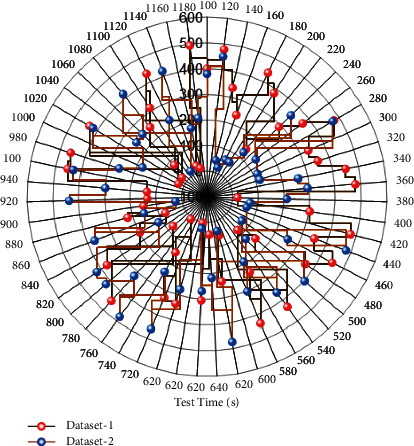
System identification results.

In entering the data original network structure page, divided into two parts of network structure display for homogeneous network data and heterogeneous network data, the operation process is the same, first, the data set file is read, the id is used as a tag, the nodes and edges of the data set are obtained, nodes and edges are added, the graph size is set, a random distribution graph of nodes is drawn, the style of points and edges is set, the chart to a binary file is converted, then it is converted it to an HTML file to realize passing the graph to the front-end, connecting to the database, getting the data set introduction in the data set table, setting the parameters, passing the parameters to the front-end, returning the data set original network graph display page, and by completing the deployment of the urban old space prototype system in the Jetson Nano module component, the image recognition can be started [28]. In this article, the image data that need to be recognized output will be uploaded to the urban old space detection prototype system. The system will call the prototype system program that has been burned into the Micro SD card, which has already made the call of the urban old space recognition model, so only the images need to be uploaded to the prototype system. The identification results of the Jetson Nano component urban old space prototype system are shown in Figure 8.

**Figure 9:**
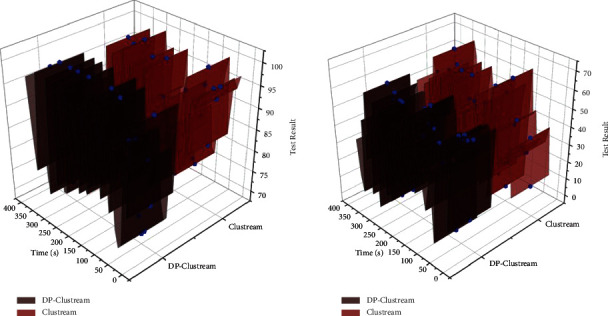
Comparison of data from multidimensional visual reconstruction system.

An exploratory visualization system with an easy-to-understand interface and component-based packaging can be used for old urban spaces to analyze multidimensional data quickly. Dynamic interaction is added with data charts and professional solutions are provided for the flexible generation of complex chart configurations and relaxed data interaction. The display range of charts is specified for small-scale zoom loading. The charts are linked to each other and can foreshadow the hidden data relationships in the same table. Dynamic and complex data chart interaction solves the problem of monolithic charting in traditional visualization systems. It ensures real-time updating of data charts and real-time monitoring of data, and changes in data sources lead to automatic updating of visualization charts, solving the problem of unchanging charts in the system due to changes in multidimensional data anytime and anywhere, and reducing the steps of redrawing diagrams due to data changes for old urban spaces. In summary, the exploratory multidimensional analysis and visualization system can sufficiently support the request of the ancient metropolitan area, good data interface interaction reaches the original intention of the design, the test results all meet the expectation. Combined with the characteristics of multidimensional data, it solves the bottleneck in the system and improves the mastery of the actual spatial data of the old urban area. The data comparison of the multifaceted visual reconstruction system is shown in Figure 9.

## 6. Conclusion

The gradual transformation of the urban economic model has brought about a change in the way of urban construction, from the previous incremental planning to the gradual transition of stock optimization. For the old urban space, using the means of organic renewal to promote urban development is the primary means of urban stock renewal. When using deep convolutional networks to extract buildings in high-resolution remote sensing images, fully convolutional networks with jump-connected structures have semantic and spatial inconsistency problems when fusing high-level features and underlying features. In this study, the attention reweighting technique is applied to embed the joint attention module in the jump structure part of the U-shaped network structure (U-Net), which can effectively adjust and optimize the underlying features from the semantic channel and spatial dimension so that the finally obtained fused elements have both intense semantic expressiveness and spatial details. The current visualization system lacks support for data functions such as multidimensional data integration, data merging table processing, multifaceted data layering, etc. It does not support data chart synergy changes well for multifaceted data. Although there are many third-party data sources, the speed of data processing is slow, which indirectly affects the results of data analysis. This study designs and implements a web-based big data exploratory multidimensional analysis and visualization system, curating a complete set of multidimensional data analysis architecture from data access to visualization display; on this basis, it develops a big data exploratory multidimensional analysis and visualization system with five modules of data source import, data processing and modeling, visualization of multidimensional data, exploration of interactive views, and dynamic combination of ideas, providing multidimensional visualization construction services with data processing and view exploration interactive application capabilities.

## Data Availability

The data used to support the findings of this study are available from the corresponding author upon request.

## References

[1] Chen X., Wang H. H., Tian B. (2019). Visualization model of big data based on self-organizing feature map neural network and graphic theory for smart cities. *Cluster Computing*.

[2] Chen M., Ludtke S. J. (2021). Deep learning-based mixed-dimensional Gaussian mixture model for characterizing variability in cryo-EM. *Nature Methods*.

[3] Peng L. (2021). Intelligent landscape design and land planning based on neural network and wireless sensor network. *Journal of Intelligent and Fuzzy Systems*.

[4] Meng N., So H. K. H., Sun X., Lam E. Y. (2019). High-dimensional dense residual convolutional neural network for light field reconstruction. *IEEE Transactions on Pattern Analysis and Machine Intelligence*.

[5] Njitacke Z. T., Isaac S. D., Nestor T., Kengne J. (2021). Window of multistability and its control in a simple 3D Hopfield neural network: application to biomedical image encryption. *Neural Computing & Applications*.

[6] Ye S., Chen Z., Chu X. (2020). Shuttlespace: exploring and analyzing movement trajectory in immersive visualization. *IEEE Transactions on Visualization and Computer Graphics*.

[7] Li L., Yan J., Wang H., Jin Y. (2020). Anomaly detection of time series with smoothness-inducing sequential variational auto-encoder. *IEEE Transactions on Neural Networks and Learning Systems*.

[8] Erol B., Amin M. G. (2019). Radar data cube processing for human activity recognition using multisubspace learning. *IEEE Transactions on Aerospace and Electronic Systems*.

[9] Motta S., Pandini A., Fornili A., Bonati L. (2021). Reconstruction of ARNT PAS-B unfolding pathways by steered molecular dynamics and artificial neural networks. *Journal of Chemical Theory and Computation*.

[10] Osia S. A., Shahin Shamsabadi A., Sajadmanesh S. (2020). A hybrid deep learning architecture for privacy-preserving mobile analytics. *IEEE Internet of Things Journal*.

[11] Kuan A. T., Phelps J. S., Thomas L. A. (2020). Dense neuronal reconstruction through X-ray holographic nano-tomography. *Nature Neuroscience*.

[12] Williams H. J., Taylor L. A., Benhamou S. (2020). Optimizing the use of biologgers for movement ecology research. *Journal of Animal Ecology*.

[13] Zhang X., Gao Y., Lin J., Lu C.-T. Tapnet: multivariate time series classification with attentional prototypical network.

[14] Simon L. M., Wang Y.-Y., Zhao Z. (2021). Integration of millions of transcriptomes using batch-aware triplet neural networks. *Nature Machine Intelligence*.

[15] Ismail Fawaz H., Forestier G., Weber J., Idoumghar L., Muller P.-A. (2019). Deep learning for time series classification: a review. *Data Mining and Knowledge Discovery*.

[16] Wang Y., Yan G., Zhu H. (2020). VC-Net: deep volume-composition networks for segmentation and visualization of highly sparse and noisy image data. *IEEE Transactions on Visualization and Computer Graphics*.

[17] Buhrmester V., Münch D., Arens M. (2021). Analysis of explainers of black box deep neural networks for computer vision: a survey. *Machine Learning and Knowledge Extraction*.

[18] Cao M., Zheng L., Jia W., Liu X. (2020). Joint 3D reconstruction and object tracking for traffic video analysis under IoV environment. *IEEE Transactions on Intelligent Transportation Systems*.

[19] El Sayad Z. T., Ayad H. M. (2019). VRGIS as assistance tool for urban decision making: rafah–Gaza–Palestine. *Alexandria Engineering Journal*.

[20] Cai D. (2019). Ecological study of the application of flowers plant real-time observation and 3D reconstruction based on kinect. *Ekoloji*.

[21] Wang X., Wang K., Lian S. (2020). A survey on face data augmentation for the training of deep neural networks. *Neural Computing & Applications*.

[22] Soffer S., Ben-Cohen A., Shimon O., Amitai M. M., Greenspan H., Klang E. (2019). Convolutional neural networks for radiologic images: a radiologist’s guide. *Radiology*.

[23] Zhu D., Song D., Chen Y. Deep unsupervised binary coding networks for multivariate time series retrieval.

[24] Waxenegger-Wilfing G., Dresia K., Deeken J. C., Oschwald M. (2020). Heat transfer prediction for methane in regenerative cooling channels with neural networks. *Journal of Thermophysics and Heat Transfer*.

[25] Chang Y., Chen M., Yan L., Zhao X.-L., Li Y., Zhong S. (2019). Toward universal stripe removal via wavelet-based deep convolutional neural network. *IEEE Transactions on Geoscience and Remote Sensing*.

[26] Asadi A., Arefi H., Fathipoor H. (2020). Simulation of green roofs and their potential mitigating effects on the urban heat island using an artificial neural network: a case study in Austin, Texas. *Advances in Space Research*.

[27] Catak F. O., Mustacoglu A. F. (2019). Distributed denial of service attack detection using autoencoder and deep neural networks. *Journal of Intelligent and Fuzzy Systems*.

[28] Elton D. C., Boukouvalas Z., Fuge M. D., Chung P. W. (2019). Deep learning for molecular design-a review of the state of the art. *Molecular Systems Design & Engineering*.

